# Redox Signaling and Myocardial Cell Death: Molecular Mechanisms and Drug Targets

**DOI:** 10.1155/2016/3190753

**Published:** 2016-07-03

**Authors:** Mohanraj Rajesh, Lu Cai, Partha Mukhopadhyay, Srinivasan Vedantham

**Affiliations:** ^1^Department of Pharmacology and Therapeutics, College of Medicine and Health Sciences, United Arab Emirates University, Al Ain 17666, UAE; ^2^Kosair Children's Hospital Research Institute and Departments of Pediatrics, Radiation Oncology and Pharmacology and Toxicology, University of Louisville, Louisville, KY 40202, USA; ^3^Laboratory of Cardiovascular Physiology and Tissue Injury, National Institute on Alcohol Abuse and Alcoholism, National Institutes of Health, Bethesda, MD 20892-2413, USA; ^4^School of Chemical Biotechnology, Department of Biotechnology, SASTRA University, Thanjavur 613401, India

Cardiovascular diseases (CVD) are the major causes of mortality and morbidity in the world. Apoptotic cardiac cell death has been reported in myocardial tissues obtained from patients with congestive heart failure, myocardial infraction, arrhythmogenic right ventricular dysplasia, myocarditis, chemotherapy induced cardiomyopathies, diabetic cardiomyopathy, and so forth. Further, loss of functional capacity of the myocytes via apoptotic cell death accounts for the major cause of morbidity and mortality in the above-mentioned heart diseases. Thus prevention of cardiomyocyte cell death [preserving functional myocardium] could profoundly improve the clinical outcome of the treatment for the aforementioned CVD. Despite significant progress made with clinical management of CVD, development of specific inhibitors to thwart the cardiomyocyte apoptosis is currently restricted due to the limited knowledge underlying the signaling process involved in this process. In addition, the factors triggering and mediating the apoptotic cell death in the myocardium are also murky. Therefore, it is imperative to understand risk factors, regulators, and biomarkers for apoptotic cell death which could aid in the development of therapeutic strategies to mend the injured myocardium.

Myocardial ischemia/reperfusion (I/R) injury is often encountered during various surgical interventions of CVD. Although the sterile inflammation plays a pivotal role in resolving the myocardial tissue injury, dysregulated inflammation process can alter the homeostasis process and perpetuate the tissue injury. Excessive generation of reactive oxygen species (ROS) due to mitochondrial dysfunction is thought to be the central player in myocardial injury. D. M. Muntean et al. have thoroughly reviewed the various sources of ROS arising from mitochondria and their physiological and pathological role during the myocardial ischemia/reperfusion (I/R) injury. Further, the authors also discussed the therapeutic strategies and mitochondria targeted antioxidants molecules being investigated in clinical trial targeting mitochondrial dysfunction associated with I/R injury. In a similar tone, G. A. Kurian et al. in their review article have discussed the central role of oxidative, reductive stress in the development of myocardial I/R injury, diabetic heart disease, and heart failure.

Stroke accounts for the major cause of mortality among subjects with increased risk for CVD such as in patients with diabetes, hypertension, and dyslipidemia. In an effort to develop newer therapeutic agent for the management of stroke, H. Hu et al. used an active component isosteviol derived from* Stevia rebaudiana* leaf and demonstrated the cerebral-vascular protective property in a rodent model of stroke. Further, Z. Fan et al. demonstrated that isosteviol could modulate the sarcK_ATP_ and mitoK_ATP_ channels in isolated ventricular myocytes. J. Pálóczi et al. demonstrated that nitric oxide donor protects the cardiomyocytes derived from human embryonic stem cells against ischemia induced apoptosis.

Geriatric population is at increased risk of developing sepsis. F. Li et al. demonstrated the key role of translocation factor EB (TFEB) in regulating the lipopolysaccharide induced inflammation, oxidative stress, autophagy, and apoptosis in the aged heart and postulated TFEB as drug target to ameliorate the myocardial tissue injury in aged subjects. Several anticancer agents exhibit profound noxious effects on the heart and increase the mortality rates among cancer survivors. Therefore, myocardial toxicity imposed by these anticancer agents often restricts the clinical usage particularly among pediatric patients. In this direction, S. Ojha et al. have in depth reviewed the need for effective cardioprotective adjuvants that could circumvent the chemotherapeutic agent induced cardiotoxicity. Specific phytochemicals and their mechanistic actions in combating doxorubicin induced cardiotoxicity in various preclinical studies were discussed. Burn can inflict severe myocardial tissue injury. W. Cai et al. demonstrated the pivotal role of Notch signaling pathway in mitigating burn-induced myocardial tissue in a rodent model.

The articles published in this special issue have addressed some of the contentious issues that pertain to redox signaling and myocardial cell death in CVD. We hope that these articles could stimulate our continuing efforts to understand the molecular and cellular pathophysiological mechanisms and impairments that culminate in the cardiomyocyte death in CVD.

*Mohanraj Rajesh* *Mohanraj Rajesh* *Lu Cai* *Lu Cai* *Partha Mukhopadhyay* *Partha Mukhopadhyay* *Srinivasan Vedantham* *Srinivasan Vedantham*

